# Mycoplasma pneumoniae Infection Associated With a Severe Asthma Exacerbation in a Five-Year-Old Child: A Case Report

**DOI:** 10.7759/cureus.117658

**Published:** 2026-10-08

**Authors:** Asmae Yahia, Fatima Zahra Alaoui-Inboui, Othmane Ahmito, Kamilia Chbani, Bouchra Slaoui

**Affiliations:** 1 Pediatrics, Pneumo-Allergology Unit, Abderrahim Harouchi Children's Hospital, Ibn Rochd University Hospital Center, Casablanca, MAR; 2 Pediatric Radiology, Abderrahim Harouchi Children's Hospital, Ibn Rochd University Hospital Center, Casablanca, MAR

**Keywords:** asthma exacerbation, child, macrolides, mycoplasma pneumoniae, respiratory infection

## Abstract

*Mycoplasma pneumoniae* is a recognized respiratory pathogen in children and may act as a trigger for acute asthma exacerbations. We report the case of a five-year-old child with known asthma and Down syndrome who was admitted with a severe asthma exacerbation associated with marked respiratory distress and hypoxemia. Initial treatment included inhaled salbutamol, systemic corticosteroids, oxygen support, and empirical antibiotic therapy with amoxicillin, but the clinical response was slow, with persistent fever and hypoxemia. Multiplex respiratory polymerase chain reaction (PCR) testing of a nasopharyngeal sample identified *M. pneumoniae*. A macrolide was subsequently introduced, and clinical improvement was observed thereafter, alongside continued standard asthma therapy, with resolution of respiratory distress, normalization of pulmonary auscultation, and recovery of oxygen saturation on room air. The present case highlights the importance of considering *M. pneumoniae* in severe or unusually prolonged asthma exacerbations, particularly when fever and hypoxemia persist despite standard asthma management.

## Introduction

*Mycoplasma pneumoniae* (*M. pneumoniae*) has been recognized as a potential infectious trigger of asthma exacerbations in children. Acute *M. pneumoniae* infection has been reported more frequently in children presenting with acute asthma exacerbations than in children with stable asthma or control subjects, suggesting a possible role in triggering acute episodes [1]. Similar findings have been reported in other pediatric populations, with evidence of recent *M. pneumoniae* infection being more frequent during acute asthma attacks than in patients with stable asthma or healthy controls [2]. Molecular studies have further supported this association, with *M. pneumoniae* detected by polymerase chain reaction (PCR) in children with asthma but not in the control group [3].

In children with asthma, persistent fever, hypoxemia, or an insufficient response to standard asthma therapy may raise suspicion of an infectious trigger, including atypical pathogens such as *M. pneumoniae*. In the present case, the coexistence of Down syndrome was considered an additional relevant clinical factor in the overall respiratory context.

We report the case of a five-year-old child with asthma and Down syndrome who presented with a severe asthma exacerbation associated with persistent fever and hypoxemia. *M. pneumoniae* infection was identified by multiplex respiratory PCR.

## Case presentation

The patient was a five-year-old child hospitalized for the management of a severe asthma exacerbation. He had been followed since the age of one year for asthma and was receiving maintenance therapy with 125 µg fluticasone propionate, one inhalation twice daily. The child had dysmorphic features consistent with Down syndrome, with no associated cardiac abnormalities identified on echocardiography. He also had an atopic background characterized by allergic rhinitis and a family history of asthma in a sibling. He had previously been hospitalized at the age of three years for a moderate asthma exacerbation. Vaccinations were up to date according to the Moroccan National Immunization Program. The current respiratory symptoms and fever had started two days before admission.

On admission, the child was alert and febrile at 38.1°C. He had severe respiratory distress with intercostal and suprasternal retractions, nasal flaring, and thoracoabdominal paradoxical breathing. Pulmonary auscultation revealed bilateral wheezing and crackles, with wheezing audible from a distance. The respiratory rate was 38 breaths/min, and the heart rate was 117 beats/min. Oxygen saturation was 84% on room air. Chest radiograph showed bronchial wall thickening, bilateral basal atelectasis, and thoracic distension (Figure 1). These radiographic findings were nonspecific and could be observed in asthma exacerbation; bronchial wall thickening may also occur in *M. pneumoniae* respiratory infection. Therefore, the radiographic findings alone were not considered diagnostic of *M. pneumoniae* infection.

**Figure 1:**
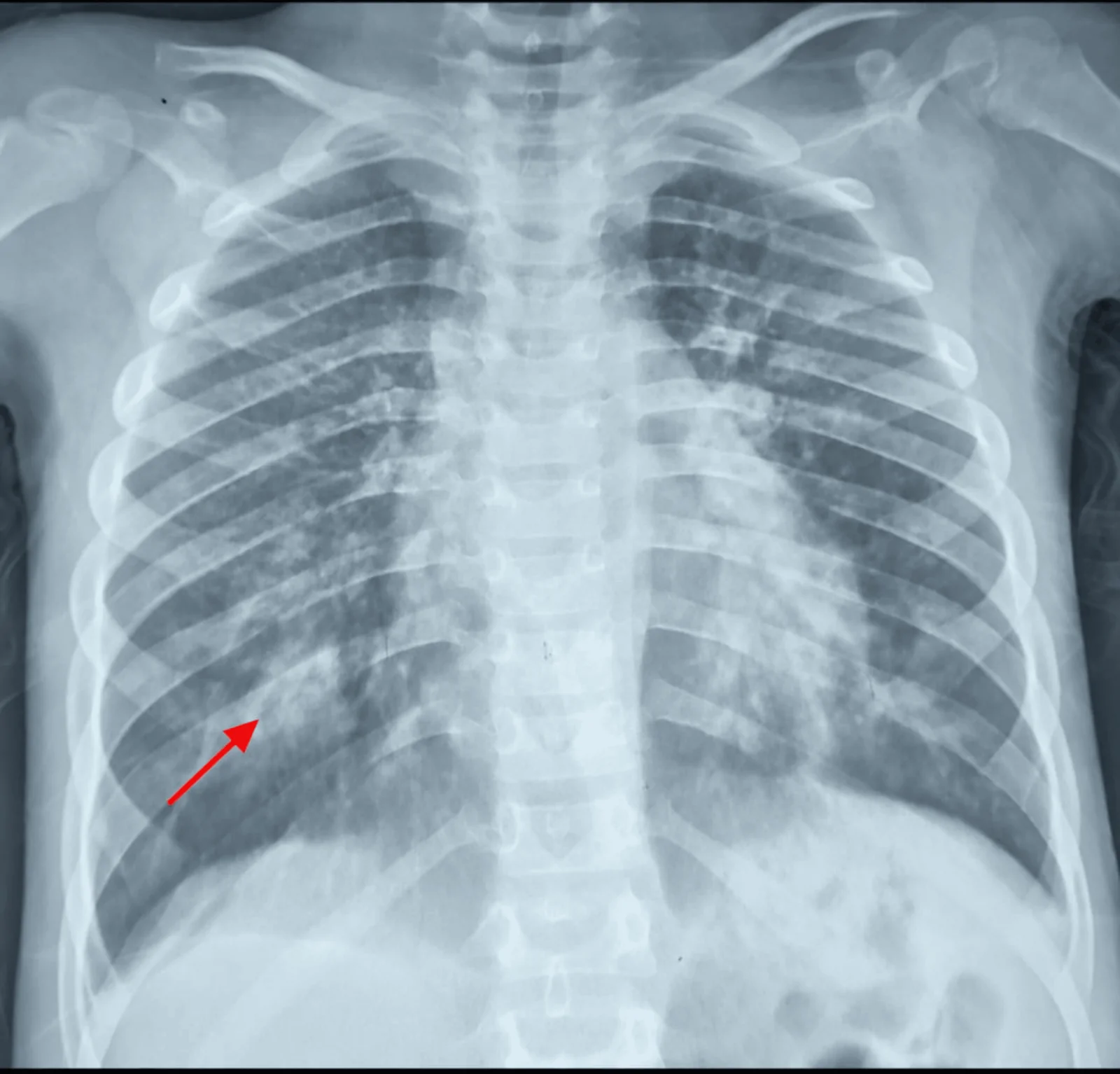
Chest radiograph at admission Chest radiograph showing bronchial wall thickening, bilateral basal atelectasis, and thoracic distension. The arrow indicates the area of right lower lobe atelectasis.

Laboratory investigations revealed leukocytosis with marked neutrophilia, lymphopenia, mild anemia, and an elevated C-reactive protein level (Table 1).

**Table 1:** Laboratory findings at admission

Laboratory parameter	Patient value	Reference range
White blood cell count (×10³/µL)	14.36	4.00-10.00
Hemoglobin (g/dL)	11.48	12.00-18.00
Platelet count (×10³/µL)	294.6	150-450
Absolute neutrophil count (×10³/µL)	13.04	1.10-7.00
Absolute lymphocyte count (×10³/µL)	0.78	1.00-5.00
C-reactive protein (mg/L)	85.7	0-10

Oxygen therapy was initiated on admission and maintained for six days. Initial management consisted of two cycles of salbutamol nebulizations (1 mL + 3 mL of normal saline) with progressively increased intervals between sessions, and systemic corticosteroid therapy with intravenous methylprednisolone at 2 mg/kg every six hours. Empirical antibiotic therapy with amoxicillin was also initiated at 100 mg/kg/day. Maintenance therapy for asthma was optimized with a fluticasone 50 µg/salmeterol combination, two puffs twice daily.

Given the persistence of severe hypoxemia and fever, as well as slow clinical improvement despite appropriate management, a multiplex respiratory polymerase chain reaction (PCR) was performed on a nasopharyngeal sample on hospital day 4. The FilmArray respiratory panel (BioFire Diagnostics; Salt Lake City, Utah, USA) was used to detect *M. pneumoniae* (Table 2). Serological testing for *M. pneumoniae*, including IgM, was not performed. Although the lymphopenia raised the possibility of a concurrent viral infection, no viral target was detected on the nasopharyngeal multiplex respiratory PCR panel. No other bacterial target included in the nasopharyngeal respiratory panel was detected.

**Table 2:** Multiplex respiratory PCR findings PCR: polymerase chain reaction

Parameter	Finding
Test	Multiplex PCR by syndromic approach-respiratory panel
Kit	FilmArray respiratory panel
Specimen	Nasopharyngeal sample
Result	Presence of bacterial targets
Detected target	Mycoplasma pneumoniae
Interpretation	Detection and identification of *Mycoplasma pneumoniae *genome

Azithromycin was initiated on hospital day 3 and administered orally at 10 mg/kg once daily for five days. Clinical improvement was subsequently observed, with resolution of respiratory distress, normalization of pulmonary auscultation, and oxygen saturation reaching 96% on room air. The child was discharged after eight days of hospitalization.

The main clinical events and therapeutic interventions are summarized in Table 3.

**Table 3:** Clinical timeline of the case PCR: polymerase chain reaction; SpO₂: peripheral oxygen saturation

Clinical stage	Key events
Admission	Severe asthma exacerbation with respiratory distress, fever, and hypoxemia (SpO₂ 84%); symptoms had started two days before admission
Initial management	Oxygen therapy, salbutamol nebulizations, intravenous methylprednisolone, and empirical antibiotic therapy with amoxicillin
Persistent symptoms	Persistent fever and hypoxemia with slow clinical improvement despite initial management
Macrolide therapy	Hospital day 3: azithromycin 10 mg/kg orally once daily for five days was initiated
Microbiological investigation	Hospital day 4: nasopharyngeal multiplex respiratory PCR detected *M. pneumoniae*
Clinical evolution	Progressive improvement in respiratory distress and pulmonary auscultation; SpO₂ reached 96% on room air
Discharge	Discharged after eight days of hospitalization

## Discussion

*Mycoplasma pneumoniae* infection is now recognized as a factor capable of triggering or worsening asthma exacerbations in children. Several studies have demonstrated a significant association between this atypical pathogen and acute manifestations of asthma.

In a prospective French study conducted in Paris, Biscardi et al. showed that acute *M. pneumoniae* infection was found in 20% of children with known asthma hospitalized for a severe exacerbation, and in 50% of those presenting for a first asthma attack [1].

This underscores the potential role of this bacterium in triggering exacerbations and even in revealing the disease in some patients. Similarly, in a Turkish study from Adana, Annagür et al. reported that 8.1% of children hospitalized for an asthma attack had positive IgM antibodies against *M. pneumoniae*, a frequency significantly higher than that observed in stable asthmatic children and healthy controls (p = 0.031) [2]. In an Indian study including 150 children, Varshney et al. found a positive PCR for *M. pneumoniae* in 15 asthmatic children, whereas none of the controls tested positive (p = 0.002), suggesting the involvement of this microorganism, particularly in moderate exacerbations [3]. Finally, in a German study, Teig et al. detected *M. pneumoniae* by PCR in 10.5% of children with chronic respiratory diseases, while none of the healthy controls were positive. This positivity was more frequent in children with non-atopic asthma, suggesting that persistent infection or prolonged colonization could contribute to the maintenance of bronchial inflammation in a subset of patients, although a formal causal link could not be established [4].

The pathophysiological mechanisms underlying this association are now better understood.

Following its adhesion to the respiratory epithelium via specific adhesion proteins, *M. pneumoniae* induces an intense inflammatory response characterized by the activation of macrophages and bronchial epithelial cells, leading to the release of pro-inflammatory cytokines such as interleukin (IL)-1β, IL-6, IL-8, and tumor necrosis factor (TNF)-α [5,6]. The infection also promotes a polarization of the immune response toward a Th2 profile, with increased production of IL-4, IL-5, IL-13, and IgE, favoring eosinophil recruitment, bronchial hyperreactivity, and mucus hypersecretion [5,7]. The community-acquired respiratory distress syndrome (CARDS) toxin, the main virulence factor of *M. pneumoniae*, plays a central role in this inflammatory response by inducing respiratory epithelial damage, mucous metaplasia, and airway hyperreactivity [5,8]. Furthermore, several studies suggest that *M. pneumoniae* can persist in the respiratory tract, sustaining low-grade chronic inflammation that may contribute to airway remodeling and the progression of asthma in genetically predisposed individuals [6,7]. In the present case, the persistence of fever and hypoxemia, along with incomplete improvement under conventional treatment for asthma exacerbation, prompted the performance of a multiplex respiratory PCR, which identified an *M. pneumoniae* infection. This atypical clinical course is consistent with the literature, which recommends considering atypical pathogen infection in any severe or prolonged exacerbation, particularly when accompanied by systemic signs or when the response to standard asthma therapy is insufficient [1,6].

Management relies primarily on the conventional treatment of asthma exacerbation, including oxygen therapy when necessary, short-acting inhaled β2-agonists, early administration of systemic corticosteroids in moderate to severe forms, and optimization of maintenance asthma therapy in accordance with international guidelines. When *M. pneumoniae* infection is confirmed or strongly suspected, macrolides, particularly azithromycin and clarithromycin, are generally considered first-line antibacterial agents in children [6,9]. Beyond their antibacterial activity, macrolides possess several immunomodulatory properties, reducing the production of pro-inflammatory cytokines, neutrophil recruitment, and mucus hypersecretion, which may contribute to more rapid clinical improvement in some patients [8,10]. However, the growing emergence of macrolide-resistant strains, notably in Asia but also in several European countries, requires judicious use of this antibiotic therapy, ideally guided by microbiological results and clinical context [9,11,12]. In cases of documented resistance or lack of clinical improvement, tetracyclines may be considered in children over eight years of age, while fluoroquinolones remain reserved for exceptional situations due to their potential adverse effects in children [9,11]. In our case, clinical improvement was observed after the initiation of azithromycin; however, a direct causal relationship cannot be established, as macrolide therapy coincided with the ongoing systemic corticosteroid treatment and optimization of maintenance asthma therapy with fluticasone/salmeterol. Therefore, the favorable clinical course likely reflected the combined effects of standard asthma management and treatment of the documented *M. pneumoniae* infection.

This case report has several limitations. As a single-patient observation, it cannot establish a causal relationship between* M. pneumoniae* infection and the asthma exacerbation or between azithromycin therapy and clinical improvement. Moreover, azithromycin initiation coincided with the ongoing systemic corticosteroid therapy and intensification of maintenance asthma treatment, limiting attribution of the favorable outcome to any single intervention. Longer-term follow-up of asthma control was not included in this report.

## Conclusions

This clinical case emphasizes the necessity of considering *M. pneumoniae* infection in cases of severe or atypical asthma exacerbation in children, particularly in the presence of persistent fever or an insufficient response to conventional treatment. Multiplex respiratory PCR enables early diagnosis and guides appropriate management. In this case, clinical improvement occurred following the combination of standard asthma management and macrolide therapy for documented *M. pneumoniae* infection; however, the individual contribution of azithromycin to the favorable outcome cannot be determined.
